# Presence of S100A9-positive inflammatory cells in cancer tissues correlates with an early stage cancer and a better prognosis in patients with gastric cancer

**DOI:** 10.1186/1471-2407-12-316

**Published:** 2012-07-28

**Authors:** Biao Fan, Lian-Hai Zhang, Yong-ning Jia, Xi-Yao Zhong, Yi-Qiang Liu, Xiao-Jing Cheng, Xiao-Hong Wang, Xiao-Fang Xing, Ying Hu, Ying-Ai Li, Hong Du, Wei Zhao, Zhao-Jian Niu, Ai-Ping Lu, Ji-You Li, Jia-Fu Ji

**Affiliations:** 1 Department of Surgery, Key laboratory of Carcinogenesis and Translational Research (Ministry of Education), Peking University Cancer Hospital & Institute, Beijing, China; 2 Department of Pathology, Key laboratory of Carcinogenesis and Translational Research (Ministry of Education), Peking University Cancer Hospital & Institute, Beijing, China; 3 Clinical Gastric Cancer Research Laboratory, Key laboratory of Carcinogenesis and Translational Research (Ministry of Education), Peking University Cancer Hospital & Institute, Beijing, China; 4 Tissue Bank, Key laboratory of Carcinogenesis and Translational Research (Ministry of Education), Peking University Cancer Hospital & Institute, Beijing, China; 5 Clinical Research Laboratory, Key laboratory of Carcinogenesis and Translational Research (Ministry of Education), Peking University Cancer Hospital & Institute, Beijing, China; 6 Department of General Surgery, Affiliated Hospital of Medical College Qingdao University, Shandong Province, China

**Keywords:** Gastric cancer, S100A9, Inflammatory cells, Tumor staging, Survival

## Abstract

**Background:**

S100A9 was originally discovered as a factor secreted by inflammatory cells. Recently, S100A9 was found to be associated with several human malignancies. The purpose of this study is to investigate S100A9 expression in gastric cancer and explore its role in cancer progression.

**Methods:**

S100A9 expression in gastric tissue samples from 177 gastric cancer patients was assessed by immunohistochemistry. The expression of its dimerization partner S100A8 and the S100A8/A9 heterodimer were also assessed by the same method. The effect of exogenous S100A9 on motility of gastric cancer cells AGS and BGC-823 was then investigated.

**Results:**

S100A9 was specifically expressed by inflammatory cells such as macrophages and neutrophils in human gastric cancer and gastritis tissues. Statistical analysis showed that a high S100A9 cell count (> = 200) per 200x magnification microscopic field in cancer tissues was predictive of early stage gastric cancer. High S100A9-positive cell count was negatively correlated with lymph node metastasis (*P* = 0.009) and tumor invasion (*P* = 0.011). S100A9 was identified as an independent prognostic predictor of overall survival of patients with gastric cancer (*P* = 0.04). Patients with high S100A9 cell count were with favorable prognosis (*P* = 0.021). Further investigation found that S100A8 distribution in human gastric cancer tissues was similar to S100A9. However, the number of S100A8-positive cells did not positively correlate with patient survival. The inflammatory cells infiltrating cancer were S100A8/A9 negative, while those in gastritis were positive. Furthermore, exogenous S100A9 protein inhibited migration and invasion of gastric cancer cells.

**Conclusions:**

Our results suggested S100A9-positive inflammatory cells in gastric cancer tissues are associated with early stage of gastric cancer and good prognosis.

## Background

Gastric cancer is one of leading causes of cancer mortality worldwide. A total of 989,600 new stomach cancer cases and 738,000 deaths were estimated to have occurred in 2008, and over 70% of new cases and deaths occur in developing countries such as China [1]. Gastric cancer is commonly detected at advanced stages, when prognostic outcomes are poor. Nearly 70-80% of patients have involvement of the regional lymph nodes which has a profound influence on survival [2,3]. Therefore, discovery of new biomarkers aiding in early detection and accurate prediction of tumor behavior could improve patient survival [4-6].

Members of the S100 family of proteins are emerging as biomarkers in multiple types of tumors [7]. The S100 family member S100A9 is a 13kd protein that contains conserved structural motifs consisting of two EF-hand Ca^2+^-binding domains. After calcium binding, S100A9 interacts with another S100 family member S100A8 to form the functional heterodimer called calprotectin [8,9]. S100A9 was originally identified as a factor secreted by inflammatory cells such as neutrophils and macrophages in rheumatoid arthritis, inflammatory bowel disease, and other inflammatory diseases [10-14]. S100A9, S100A8, as well as the S100A8/A9 heterodimer calprotectin, are overexpressed during inflammation-induced carcinogenesis [15]. S100A9 expression is up-regulated in tumor cells in lung [16], prostate [17], and breast cancer [18,19], while it is down-regulated in human esophageal cancer cells [20]. In colorectal cancer tissue specimens, however, the S100A9 protein was not detected in cancer cells, but rather in inflammatory cells scattered throughout the tumor stroma [21]. In addition, S100A9 was significantly higher in stool samples of colorectal cancer patients than in controls [22]. In gastric cancer, gene expression and proteomic analysis demonstrated high expression of S100A9 in the tissue. [23,24]. However, its distribution within the tissue and association with clinicopathological features were not fully demonstrated.

In this study, we used gene expression analysis to compare S100A9 expression in gastric cancer tissues and in the adjacent, ostensibly normal tissues. Immunohistochemical staining revealed S100A9 in tumor-associated inflammatory cells. Furthermore, we addressed the correlation between the number of S100A9-positive cells in tumor tissues and the clinicopathological features. We also addressed the co-localization of S100A9 and S100A8 as well as the localization of the dimer calprotectin by immunofluorescence. Finally, to gain insight into the function of S100A9 in cancer cells, we investigated the effect of the recombinant S100A9 protein on migration and invasion of gastric cancer cells AGS and BGC-823.

## Methods

### Patients and tissue specimens

This investigation was performed after approval by Ethics Committee of Peking University Cancer Hospital. Informed consent was obtained from each patient. One hundred seventy-six patients with gastric cancer were studied. 124 males and 53 females (mean age, 57 years; range, 26–80 years) were diagnosed and surgically treated in Peking University Cancer Hospital between 1998 and 2004. The depth of tumor invasion, histological grade, lymph node metastasis, liver metastasis, and vascular invasion were obtained from clinical and histopathological reports. Stage of gastric cancer was classified according to the 7th edition tumor-node metastasis (TNM) classification recommended by the American Joint Committee on Cancer. None of the patients received chemotherapy or radiation therapy preoperatively. All patients were followed up until January 2010. After gastrectomy, one part of resected specimen was fixed in 10% formalin and processed routinely for pathological assessment, and another was snap-frozen in liquid nitrogen stored at −80°C for RNA extraction. In addition, 30 matched metastatic lymph nodes were also collected from these patients. Ten cases of chronic appendicitis tissues with exacerbation were provided by the Department of General Surgery, the affiliated hospital of Qingdao University Medical College.

### Immunohistochemistry (IHC)

Four-micrometer sections from formalin-fixed paraffin-embedded tissues were mounted on poly-L-lysine-coated slides and then deparaffinized in xylene and rehydrated through alcohol to distilled water. Endogenous peroxidase activity was blocked with 3% hydrogen peroxide for 15 minutes at room temperature. After pressure cooking the slides in 10 mmol/L EDTA (pH 8.0) for 3 minutes, the sections were incubated with 5% goat serum, then incubated overnight at 4°C with mouse anti-S100A9 antibody (1:200, T1028, BMA Biomedicals, Switzerland), or mouse anti-S100A8 antibody (1:200, T1031, BMA Biomedicals), or mouse anti-S100A8/A9 antibody (1:200, T1023, BMA Biomedicals). Primary antibodies were detected using a two-step EnVision System (Dako, Glostrup, Denmark). Horseradish peroxidase and diaminobenzedene hydrochloride (DAB) were enzyme and chromogen employed. Expression of S100A9, S100A8 and S100A8/A9 were also detected in Cybrdi tissue microarray slides (IC00-01-001, Cybrdi, Xi'an, China) containing chronic gastritis with metaplasia (57 cases) and gastric carcinoma tissues (23 cases). Ten cases of chronic appendicitis specimens with exacerbation were served as positive control for S100A8/A9.

### IHC assessment and cut-off definition

S100A9 and S100A8 were stained in the inflammatory cells such as macrophages and neutrophils infiltrating tumor tissues. Positive cells showed a variable degree of cytoplasmic staining. Images were acquired using Ariol image analysis system (Applied Imaging, San Jose, CA, USA). The scanner is based on an Olympus BX61 microscope with a motorized stage and autofocus capabilities equipped with a camera. Slides were scanned at 200× magnification. The degree of monoclonal S100A9 or S100A8 antibody reactivity in each tissue section was assessed by counting the number of stained inflammatory cells in three 200× magnification scopes. This was conducted by two independent pathologists with the help of an automatic microscope system and the image processing software (see Additional file 1: Figure S1). Cut-off value of S100A9 stained inflammatory cells for the prediction of patient pathological stage was determined by receiver operating characteristic (ROC) curve.

### Laser confocal scanning

To investigate the co-localization of S100A9 and its dimerization partner S100A8, or the heterodimer S100A8/A9, the Cybrdi tissue microarray slides (IC00-01-001) were incubated 1.5 hours at room temperature with mouse anti-S100A9 antibody (1:200) pre-labeled with the Zenon Alexa Fluor 647 Mouse IgG Labeling Kit (Z-25008, red fluorescence), and either the anti-S100A8 antibody (1:200) or anti-S100A8/A9 antibody (1:200) pre-labeled with the Zenon Alexa Fluor 488 Mouse IgG Labeling Kit (Z-25002, green fluorescence). Confocal images were acquired using the Leica TCS SP5 confocal microscope (Leica, Mannheim, Germany). In addition, the nuclei counterstained with DAPI (Vector, Burlingame, CA, USA), excitation at 358 nm.

Specimens of chronic appendicitis tissues with exacerbation were incubated with anti-S100A9 antibody (1:200, red fluorescence labeled), and anti-S100A8/A9 antibody (1:200, green fluorescence labeled) as a positive control for the specific S100A8/A9 heterodimer expression.

### Cell culture

Both cell lines in this study were previously profiled by microarray analysis and were regularly verified using STR analysis (short tandem repeat DNA fingerprinting) [25]. Gastric cancer cell line AGS was obtained from ATCC (American Type Culture Collection, Manassas, VA), and cell line BGC-823 was established in China and obtained from Cell Research Institute, Shanghai, China. Cancer cells were routinely grown as a monolayer in RPMI-1640 medium (GIBCO BRL, Carlsbad, CA), supplemented with 10% (v/v) fetal calf serum (FCS, GIBCO) and antibiotics at 37°C in a humidified 5% CO_2_ atmosphere.

### Cell invasion assay

CytoSelect 24-Well Cell Invasion Assay kit was purchased from Cell Biolabs, USA. S100A9 recombinant protein was purchased from BMA Biomedicals, Switzerland. Cell invasion assays were performed with Transwell Inserts, which allows cells to migrate through an 8 μm pore size polycarbonate membrane. The upper surface of the insert membrane was coated with a uniform layer of dried basement membrane matrix solution. Cells resuspended in serum-free medium were plated in the upper chamber of each Transwell at a density of 10^6^ cells/mL (200μL/chamber). S100A9 recombinant protein was added to the upper chamber medium at 0, 10, 20, 50, or 100 ng/ml. The bottom chamber was filled with 500μL medium containing 10% FCS. Cells were allowed to migrate for 48 h at 37°C. Cells that remained in the upper chamber were removed with a cotton swab, and cells that had penetrated to the bottom side of the membrane were stained in Cell Stain Solution for 15 minutes, and counted in nine randomly selected microscopic fields (200×) per well. Each insert was then transferred to an empty well and incubated in 200μL of Extraction Solution. After 10 minutes, 100μL of solution from each sample were transferred to a 96-well microtiter plate and measured in a plate reader at OD560nm.

### Cell migration assay

Cell mobility was assessed using a wound healing assay. Cells were seeded into six-well tissue culture dishes and cultured until confluent to get a cell monolayer, which was then wounded using sterile 200 μl pipette tips. Any cellular debris was removed by washing with PBS. The wounded monolayer cell was then incubated in medium with 100 ng/ml S100A9 recombinant protein. Control cells were treated with serum-free RPMI-1640 medium. Time-lapse images were captured using an inverted phase-contrast microscope at 200× magnification for 0, 24, and 48 h. The cell migration ability was evaluated by calculating the average cell migration distance.

### Statistical analysis

Clinicopathologic variables were extracted from clinical and histopathological reports. ROC curves were used in determining the cut-off value of the S100A9-positive inflammatory cell count in evaluating pathological TNM stage. The association of S100A9-positive inflammatory cell count with different TNM stages was done with the Wilcoxon rank-sum test. To obtain associations between S100A9 or S100A8 cell count and clinicopathologic variables, the data was cross-tabulated and a *χ*^2^ test was performed. Cumulative survival was estimated with the Kaplan–Meier method, and comparisons between groups were done with a log-rank test. Overall survival was measured from date of initial surgery to date of death, counting death from any cause as the end point, or the last date of information as the end point if no event was documented. A multivariate analysis of the Cox proportional hazards regression model (backward, stepwise) was created to assess the influence of each variable on survival. Significance was set at *P* < 0.05.

## Results

### Expression of S100A9 in infiltrating inflammatory cells in gastric cancer and chronic gastritis tissues

**Figure 1:**
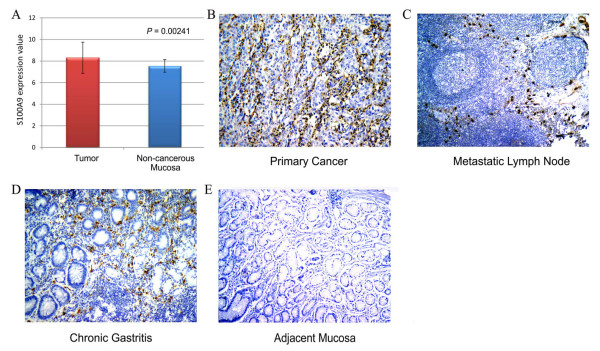
**Expression of S100A9 in gastric cancer and adjacent non-cancerous tissues.** (**A**) Different expression value of S100A9 in 72 gastric cancer tissues and paired no-cancerous tissues by analyzing data from illumina Sentrix BeadChip cDNA microarray. (B-E) Immunohistochemical staining of S100A9 in gastric cancer tissues (**B**) metastatic lymph nodes (**C**), chronic gastritis (**D**), and adjacent non-cancerous gastric mucosa (**E**). S100A9 localization was revealed as brown or red granulated loci in the cytoplasm of infiltrating inflammatory cells, especially in mononuclear phagocytes and neutrophil granulocytes. (magnification 200×).

In a previous gene array analysis, we found that gastric cancer tissues were distinguished from adjacent noncancerous mucosa by characteristic differences in their gene expression patterns [26]. The diversity of gene expression patterns may reflect variation in the intrinsic properties of tumor and normal cells as well as variation in the cellular composition of these complex tissues. Within these genes, the expression of S100A9 in gastric cancer tissues was higher than that of matched adjacent noncancerous mucosa (*P* = 0.00241, Figure 1A).

Immunohistochemistry of specimens from 177 gastric cancer patients showed that S100A9 was positive in all primary cancer tissues with immunostaining exclusively located in inflammatory cells such as macrophages and neutrophils infiltrating primary tumor tissues (The different cell types in tissue samples were identified by two independent pathologists) (Figure 1B). All examined metastatic lymph nodes (n = 30) were also positive for S100A9 with immunostaining exclusively located in inflammatory cells surrounding the metastatic cancer tissues (Figure 1C). In adjacent non-cancerous mucosa, S100A9 was expressed in inflammatory cells infiltrating gastritis. Gastric mucosa had negative or very weak S100A9 expression (Figure 1D, E).

### S100A9-positive inflammatory cell count in cancer tissues is associated with cancer stage and patient survival

**Figure 2:**
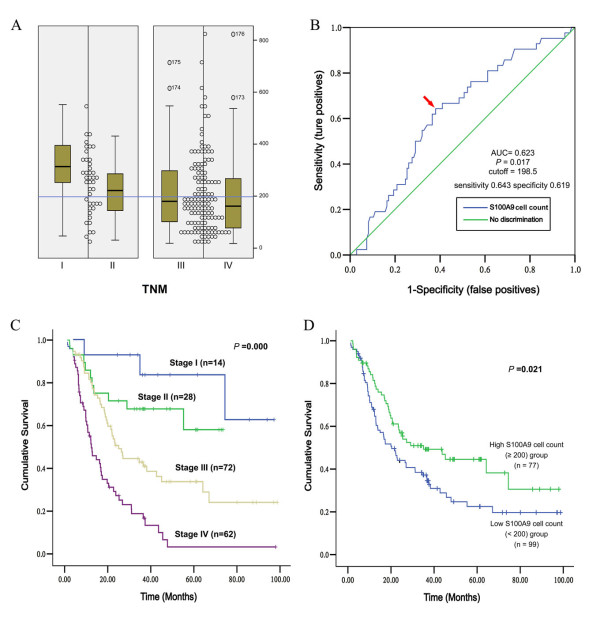
**High S100A9 cell count in cancer tissue indicates better outcome in gastric cancer patients.** (**A**) Scatter plot of S100A9-positive inflammatory cell count in each pathological TNM stage. The blue line indicates the level of 200. (**B**) ROC curve of S100A9 cell count for the prediction of pathological TNM stage. Arrow indicated the cutoff point about 200. (**C**) Kaplan-Meier analysis of overall survival for each pathological TNM stage. (**D**) Kaplan-Meier analysis of overall survival for high S100A9 cell count (≥ 200) group and low S100A9 cell count (< 200) group. (Only 176 patients were enrolled in the analysis for one patient lost to follow-up).

To evaluate the extent of S100A9 expression in gastric cancer-associated environment, the number of S100A9-positive inflammatory cells in each tumor tissue was measured by averaging the cell counts of three fields (original magnification, 200×) in the area with the greatest number of positive cells at the site of deepest tumor invasion. Correlation between cell count and clinicopathological parameters and patient survival was analyzed using Wilcoxon rank-sum test and Kaplan–Meier method. As shown in Figure 2A, gradual decrease of S100A9-positive inflammatory cell count in cancer tissues was associated with the increase of tumor pathological stage from I to IV (Wilcoxon rank sum test for 4 stages, *P* = 0.0265).

Then we tested the prediction power of the S100A9 cell count for tumor stage in gastric cancer. Based on TNM stage, patients were divided into two groups, less advanced group (stage I + II) and advanced group (stage III + IV). Area under curve (AUC) obtained from the receiver operating characteristic (ROC) curves using the S100A9-positive inflammatory cells count was 0.623 for pathological TNM stages. The cutoff value was 198.5 (we used 200 in the following analysis) per 200× magnification field with 64.3% sensitivity and 61.9% specificity for tumor stage prediction (Figure 2B). Cutoff line of 200 can be used to separate the less advanced group from the advanced group. The difference was significant between the two groups (Wilcoxon rank sum test for two groups, *P* = 0.017, Figure 2A).

Since survival analysis showed significantly different prognosis for gastric cancer patients in different cancer stages (Figure 2C), we analyzed the relationship between S100A9-positive inflammatory cells count and patient survival rate. Patients were stratified by cutoff value into high S100A9 cell count (≥ 200) group and low S100A9 cell count (< 200) group. 5-year survival rate was 44.6% in high cell count group versus 22.5% in low cell count group (*P* = 0.021, Figure 2D). Median survival time was 35.1 ± 10.8 months for the high cell count group and 20.3 ± 3.0 months for the low cell count group, respectively. Taken together, S100A9-positive inflammatory cell count in gastric cancer tissue can be used as a predictor to distinguish early stage and advanced gastric cancer with the cutoff of 200 positive cells/HPF. Presence of S100A9-positive inflammatory cells in cancer tissues correlates with a better prognosis in patients with gastric cancer.

### Low number of S100A9-positive inflammatory cells in cancer tissues positively correlates with poor clinicopathological features

Low S100A9 cell count was found to be correlated with tumor invasion depth (T stage), lymph node metastasis (N stage), and clinical TNM stage (*P* = 0.011, 0.009, and 0.002, respectively). Correlation between S100A9 cell count and other clinical features such as sex, age, tumor location, liver metastasis (M stage), vascular invasion and differentiation were not statistically significant (all *P* > 0.05) (Table 1). Additionally, multivariate analysis demonstrated N stage (*P* = 0.006), liver metastasis (*P* = 0.000), and S100A9 cell count (*P* = 0.046) to be independent factors in predicting overall survival (Table 2).

**Table 1:** Association of S100A9-positive inflammatory cell count in cancer tissues with clinicopathological parameters in gastric cancer patients

**Variables**	**Low S100A9 (positive cells <200)**	**High S100A9 (positive cells > = 200)**	***P *value**
Sex			0.125
Male	74	50	
Female	25	28	
Age			0.089
<50	32	18	
50-59	24	12	
60-69	33	33	
> = 70	10	15	
Tumor location			0.271
Cardia	26	15	
Non-cardia	73	63	
Depth of tumor invasion**			0.011
T1	3	2	
T2	7	19	
T3	69	42	
T4	20	15	
Lymph node metastasis			0.009
N0	13	23	
N1	32	30	
N2	32	17	
N3	22	8	
Liver metastasis			0.571
Negative	89	68	
Positive	10	10	
TNM stage			0.002
І	3	12	
II	37	35	
III	50	21	
IV	9	10	
Vascular invasion			0.742
Negative	38	31	
Positive	58	43	
Not recorded*	3	4	
Differentiation**			0.472
Well	2	4	
Moderately + Poorly	82	66	
Other types	13	6	
Not determined	2	2	

*Data incomplete.

** Fisher's exact test.

**Table 2:** Multivariate analysis of prognostic factors for overall survival of gastric cancer patients

**Variations**	***P***	**RR**	**CI (95%)**
Sex	0.671	1.106	0.694-1.765
Male versus female			
Age	0.179	1.148	0.938-1.405
<50			
50-59			
60-69			
> = 70			
Tumor location	0.545	0.864	0.538-1.387
Cardia *vs.* Non-cardia			
Differentiation	0.301	0.751	0.437-1.292
Well versus moderately + poorly			
Lymph node metastasis	0.006	1.841	1.196-2.835
N0 + N1 versus N2 + N3			
Depth of tumor invasion	0.1	1.756	0.897-3.435
T1 + T2 versus T3 + T4			
Liver metastasis	0	3.461	2.002-5.983
Negative versus Positive			
Vascular invasion	0.107	1.452	0.922-2.285
Negative versus Positive			
S100A9-positive inflammatory cell count	0.046	0.643	0.417-0.991
<200 versus > = 200			

RR: relative risk; CI: confidence interval.

### The expression status of S100A8 and the S100A8/A9 heterodimer in gastric cancer tissues and gastritis tissues

Chronic gastritis is a chronic gastric lesion, pathologically characterized by non-specific chronic inflammation of the gastric mucosa. The inflammatory cells in chronic gastritis are morphologically like those infiltrating primary gastric cancer tissues. In some cases, chronic gastritis even can lead to stomach cancer. Next, we further examined the expression of S100A8, a close family member of S100A9 and the heterodimerization form S100A8/A9 in both gastric cancer tissues and adjacent non-tumor chronic gastritis tissues in the gastric cancer specimens by performing immunohistochemistry. Similarly to the pattern of S100A9, S100A8 was expressed exclusively in inflammatory cells infiltrating both tumor tissues and adjacent gastritis tissues. S100A8 was not expressed in all gastric cancer cells and normal gastric mucosa.

Next, we quantified the number of S100A8-positive inflammatory cells in each tumor tissue as described earlier for S100A9 (Additional file 1: Figure S1). Surprisingly, S100A8 cell count in gastric cancer tissues did not correlate with most of clinicopathological features (Additional file 2: Table S1) or patient survival (Additional file 3: Figure S2). Moreover, expression of the heterodimerization form S100A8/A9 was not detected in any inflammatory cells infiltrating gastric cancer tissues, while some S100A8/A9 positive cells were identified in the chronic gastritis tissues (data not shown). These data indicated that the distribution of S100A9, S100A8 and S100A8/A9 might be different in human gastric cancer and chronic gastritis tissues.

**Figure 3:**
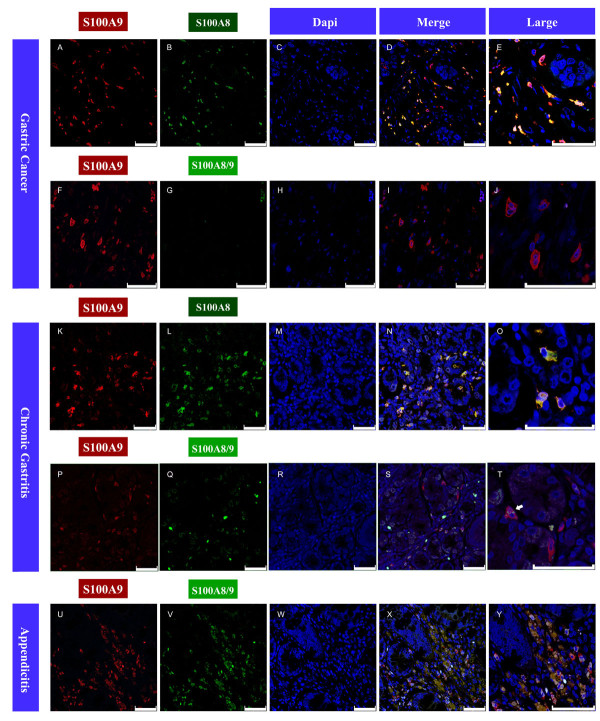
**Immunofluorescence images of S100A9, S100A8 and S100A8/A9 proteins in tissue microarray slides containing gastric cancer tissues (A-J) and chronic gastritis tissues (K-T), and chronic appendicitis tissues with exacerbation (U-Y).** S100A9 and S100A8 were detected by monoclonal antibody, prelabeled with the Zenon Alexa Fluor Mouse IgG Labeling Kit (with green and red fluorescence respectively). The nucleus was stained by DAPI. S100A8/A9 heterodimers were detectable using the dimer-specific antibody 27E10 from BMA Biomedicals prelabeled with green fluorescence. The co-localization of S100A9 and S100A8 or S100A8/A9 was showed in merged pictures (**D**, **I**, **N**, **S**, **X**) and larger merged pictures (**E**, **J**, **O**, **T**, **Y**). White arrow in 3 T shows co-localization of S100A9 and S100A8/A9 in chronic gastritis. Bar length, 50 μm.

To confirm this hypothesis, we further investigated the subcellular localization pattern of S100A9, S100A8 and S100A8/A9 heterodimer expression by performing immunofluorecence staining in a tissue microarray including 23 cases of gastric cancer and 57 cases of chronic gastritis. In gastric cancer tissues, both S100A9 and S100A8 proteins were detected in the tumor-infiltrating inflammatory cells (Figure 3A, B), while no S100A8/A9 heterodimer was found in any cases (Figure 3G). Expression of S100A8 and S100A9 partly overlapped in cytoplasm of cells (Figure 3D, E). In addition, S100A9 and S100A8 proteins were detected in inflammatory cells in chronic gastritis (Figure 3K, L). Distribution of these two proteins also partly overlapped (Figure 3N, O). Consistent with the results of immunohistochemistry, S100A8/A9 was not expressed in any cells of gastric cancer tissues (Figure 3G), while expression of S100A8/A9 partly overlapped with the S100A9 in inflammatory cells of gastritis tissues (Figure 3Q, S, T). Unsurprisingly, expression and distribution of S100A8/A9 in chronic appendicitis tissues with exacerbation (the positive control) were much alike those in chronic gastritis tissues (Figure 3U, V, X, Y). Taken together, the differential expression and subcellular localization of S100A9, S100A8 and S100A8/A9 in various tissues may implicate their different roles in gastric cancer or chronic gastritis environment.

### The inhibitory effect of the S100A9 recombinant protein on migration and invasion of gastric cancer cell lines in vitro

**Figure 4:**
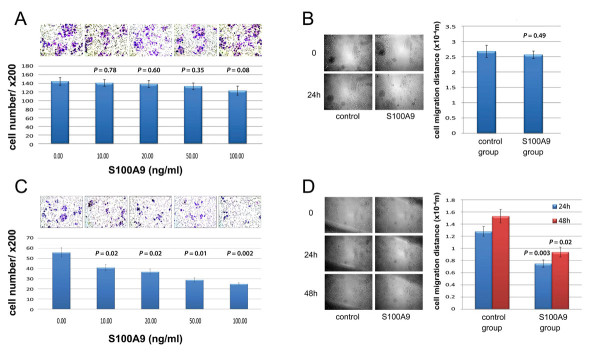
**Effect of S100A9 recombinant protein on invasion and migration of gastric cancer cell lines.** In transwell assay, AGS (**A**) and BGC-823 (**C**) cells were treated with serum-free medium or medium containing 10, 20, 50 or 100 ng/ml S100A9 recombinant protein. Invasive cells were counted in randomly nine selected microscopic fields (200×). In wound healing assay, AGS (**B**) and BGC-823 (**D**) cells were treated with serum-free RPMI-1640 medium or medium containing 100 ng/ml S100A9 recombinant protein. Photos were captured by an inverted phase-contrast microscope at 0 h, 24 h and 48 h after wounding. Three independent experiments were performed. Results were presented as means ± S.D. of these independent experiments. *P* value *vs.* control group.

The S100A9 protein is expressed in and secreted by inflammatory cells, serving as a mediator in acute and chronic inflammation. Since the S100A9-positive inflammatory cell count correlated with less aggressive clinicopathological characteristics, we further tested the direct inhibitory function of recombinant S100A9 on the migration and invasion of gastric cancer cells. To evaluate invasive ability of gastric cancer cells, transwell assays was used. Two gastric cancer cell lines, AGS and BGC-823, were treated with serum-free medium or medium containing different concentrations of S100A9 recombinant protein (10, 20, 50 and 100 ng/ml, respectively). Invasive cells were counted in nine randomly selected microscopic fields (200×). Results showed that S100A9 recombinant protein slightly inhibited AGS cell invasion (Figure 4A), while S100A9 significantly inhibited BGC-823 invasion in a concentration-dependent manner (*P* < 0.05, Figure 4C). To analyze the migrate ability of gastric cancer cells, we performed the wound healing assay. Both cell lines were treated with serum-free medium or medium containing 100 ng/ml S100A9 recombinant protein. Results showed that S100A9 slightly inhibited AGS cell migration (Figure 4B), while cell migration distances of BGC-823 cells treated with S100A9 after 24 h and 48 h of incubation were significantly lower than those of control (*P* < 0.05, Figure 4D).

## Discussion

Human cancer is a chronic disease that originates from transformed cells harboring genetic as well as epigenetic alterations. However, cancer is not composed merely of cancer cells. Cancer tissue contains other cell types, including fibroblasts and epithelial cells, immune cells, and cells forming blood vessels and lymphatic vasculature [27]. In this complex tumor microenvironment, inflammatory mediators regulate different stages of tumor development, including initiation, promotion, invasion, and metastasis [28].

S100A9, a member of S100 family, is abundant in granulocytes, monocytes and activated keratinocytes during various inflammatory conditions. In this study, we found that S100A9 was specifically located in inflammatory cells infiltrating gastric cancer tissues and chronic gastritis tissues, while all gastric cancer cells or adjacent cells of gastric mucosa did not express S100A9. Our results agree with previous studies demonstrating high expression of S100A9 in infiltrating immune cells in various cancer types including colorectal cancer [21] and pancreatic cancer [29]. At the same time, in other cancer types, such as lung cancer [16], prostate cancer [17], and breast cancer [19,30], S100A9 is expressed mainly by neoplastic tumor cells themselves. It has been suggested that tumor cells of glandular origin can express S100A9 when they are poor differentiated [18] or under pathological stress conditions [31-33]. However, we did not find any S100A9-positive gastric cancer cell including poorly differentiated ones. High expression of S100A9 in the inflammatory cells in gastric cancer tissues may indicate that S100A9 plays an important role in gastric cancer development.

Correlation among S100A9 expression, clinicopathological features and patient prognosis varies in different cancer types. In lung cancer [16] and invasive ductal carcinoma of the breast [19], overexpression of S100A9 in cancer cells has been shown to contribute to the development and progression of cancer. In thyroid carcinoma [34], expression of S100A8 and S100A9 in cancer cells is crucial for dedifferentiation. In bladder tumors, overexpression of S100A4, S100A8 or S100A11, but not S100A9 in cancer tissues is associated with stage progression, invasion, metastasis and poor survival [35]. So far studies of S100A protein expression in inflammatory cells infiltrating cancer have been rare due to lack of appropriate way to quantify the S100A expression by inflammatory cells. Only two studies evaluated S100A9 expression in a cancer-associated environment [21,29]. The results in colorectal cancer showed that high S100A9 cell count was not associated with patient survival but instead positively correlated with tumor size [21]. The other study revealed that the ratio of S100A9- and S100A8- positive cells in the stroma was affected by the status of tumor suppressor protein Smad4 in corresponding pancreatic cancer cells [29]. Our results showed that high S100A9 cell count in gastric cancer tissues was negatively correlated with advanced pathological cancer stages, lymph node metastasis, and tumor invasion. Importantly, presence of S100A9-positive inflammatory cells in cancer tissues also correlated with a better prognosis in patients with gastric cancer. In addition, the cell count of S100A8, a dimerization partner of S100A9, was not correlated with the clinicopathological features or overall survival in patients with gastric cancer. Remarkably, expression of S100A8/A9 heterodimerization complex was apparently absent in gastric cancer tissues, while it was detected in gastritis and control inflammation tissues such as appendicitis. The differential expression and subcellular localization of S100A9 and S100A8/A9 in various tissues may indicate that only S100A9 plays a role in gastric cancer development.

The association of S100A9 expression with different clinicopathological features and patient prognosis among a variety of cancer types suggests that the function of this molecule can be diverse. Growing evidence suggests that S100A9 and its close partner S100A8 and the heterodimer of S100A8/A9 can exhibit both pro- and anti-tumorigenic functions during tumorigenesis [36,37]. On one side, the up-regulation of calprotectin is a characteristic feature in pathological conditions of hyperproliferative carcinomas [38]. On the other side, several in vitro studies have demonstrated that these proteins exhibit growth-inhibitory properties as well as promote cytotoxicity and apoptosis in many cancer types [36]. Numerous studies have demonstrated the dichotomic effects of these molecules in vitro. In this study, the exogenous S100A9 recombinant protein plays a role in inhibiting the gastric cancer cell line BGC-823 migration and invasion.

The inconsistent association of S100A expression with clinicopathological features and patient prognosis among cancers may be caused by the complexity of the cancer microenvironment. Immune mediators such as S100A9 can play both anti-tumor and tumor promoting roles depending on the cancer types [39,40]. Studies have suggested that tumor promoting inflammation and anti-tumor immunity co-exist at different points along the path of tumor progression, and that environmental and microenvironmental conditions dictate the balance between the two [41,42]. Since direct in vivo models for evaluating the effects of these phenomena on cancer progression are lacking, further studies are warranted.

## Conclusions

S100A9 was specifically expressed in inflammatory cells infiltrating gastric cancer tissues and chronic gastritis tissues. Presence of S100A9-positive inflammatory cells in cancer tissues correlates with an early stage cancer and a better prognosis in patients with gastric cancer.

## Competing interests

The authors have declared that no competing interests exist.

## Authors’ contributions

J-FJ and L-HZ conceived the design of the study and were in charge of its coordination. BF, X-YZ and Y-n J carried out the immunohistochemical assays. BF, L-HZ, X-HW and X-FX performed cell line assays. Y-QL, X-JC, HD, YH, Y-AL, WZ, A-PL and J-YL participated in the clinical materials collection. BF and L-HZ conducted statistical analysis. J-FJ, BF and L-HZ drafted the manuscript. Z-JN provided chronic appendicitis tissues with exacerbation. All authors read and approved the final manuscript.

## Pre-publication history

The pre-publication history for this paper can be accessed here:

http://www.biomedcentral.com/1471-2407/12/316/prepub

## Supplementary Material

Additional file 1 **Figure S1. **To help count the inflammation cell in cancer tissues, the images under 200x magnification (**A**) were captured and processed using customized actions to automate pickup of immunostained cells (**B**) in Photoshop software (ver. 6.0). Then two pathologists counted the cells in the images (both A and B) independently. Click here for file

Additional file 2 **Table S1. ** Association of S100A8-positive inflammatory cell count in cancer tissues with clinicopathological parameters in gastric cancer patients. Click here for file

Additional file 3 **Figure S2. ** High S100A9, not S100A8 cell count indicates better outcome in gastric cancer patients. (**A**) Kaplan-Meier analysis of overall survival for high S100A8 cell count (> = 64) group and low S100A8 cell count (< 64) group in 125 gastric cancer patients. (**B**) Kaplan-Meier analysis of overall survival for high S100A9 cell count (> = 200) group and low S100A9 cell count (< 200) group in the same cohort of gastric cancer patients. Click here for file
