# Supplementary figures and images for: Presence of S100A9-positive inflammatory cells in cancer tissues correlates with an early stage cancer and a better prognosis in patients with gastric cancer
Source: BMC Cancer. 2012 Jul 28;12:316. doi: 10.1186/1471-2407-12-316 (PMC3476982; doi:10.1186/1471-2407-12-316)

Figure S1

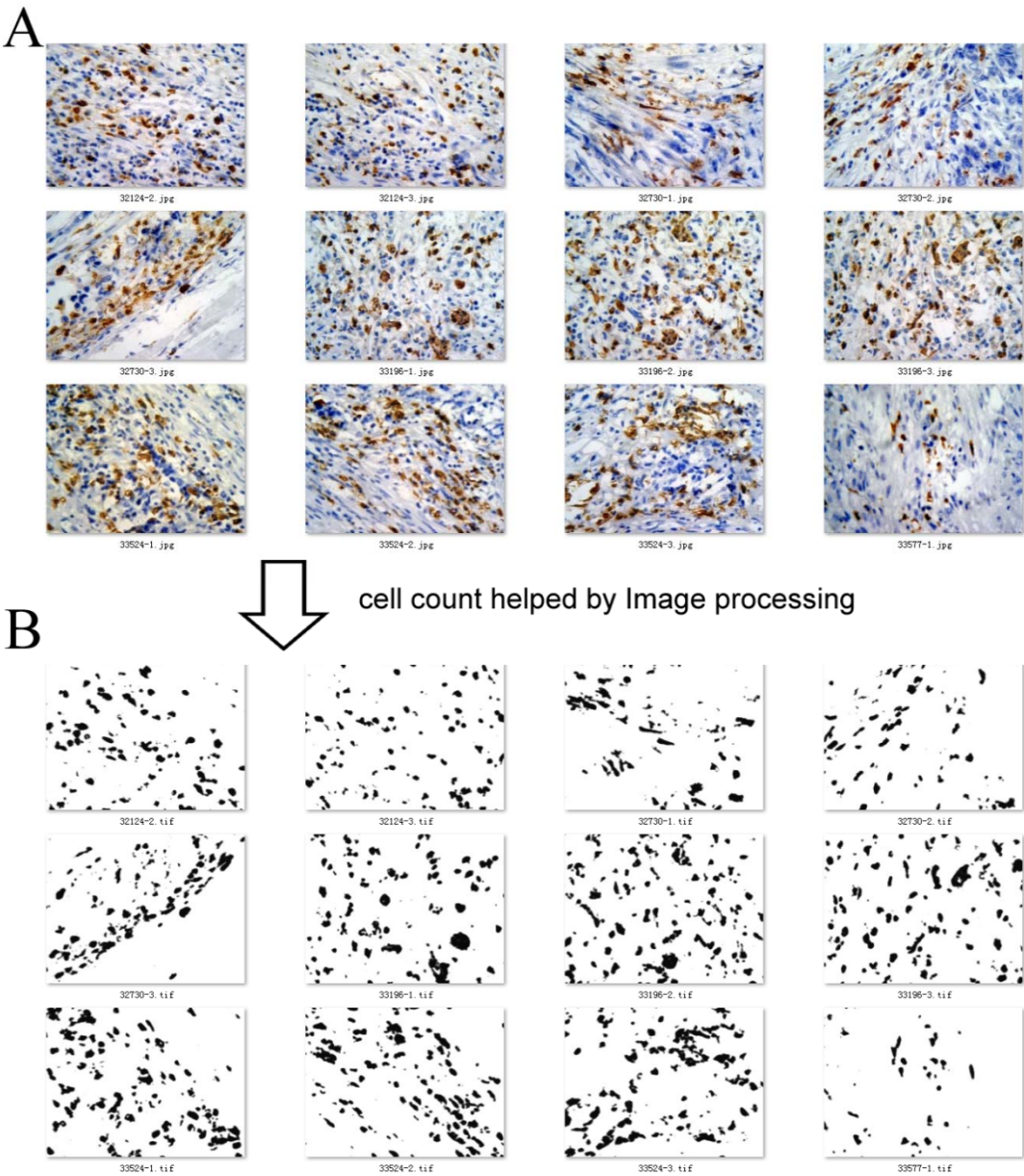

Supplement: Additional file 1 — Figure S1. To help count the inflammation cell in cancer tissues, the images under 200x magnification (A) were captured and processed using customized actions to automate pickup of immunostained cells (B) in Photoshop software (ver. 6.0). Then two pathologists counted the cells in the images (both A and B) independently. [file 1471-2407-12-316-S1.pdf]

Figure S2

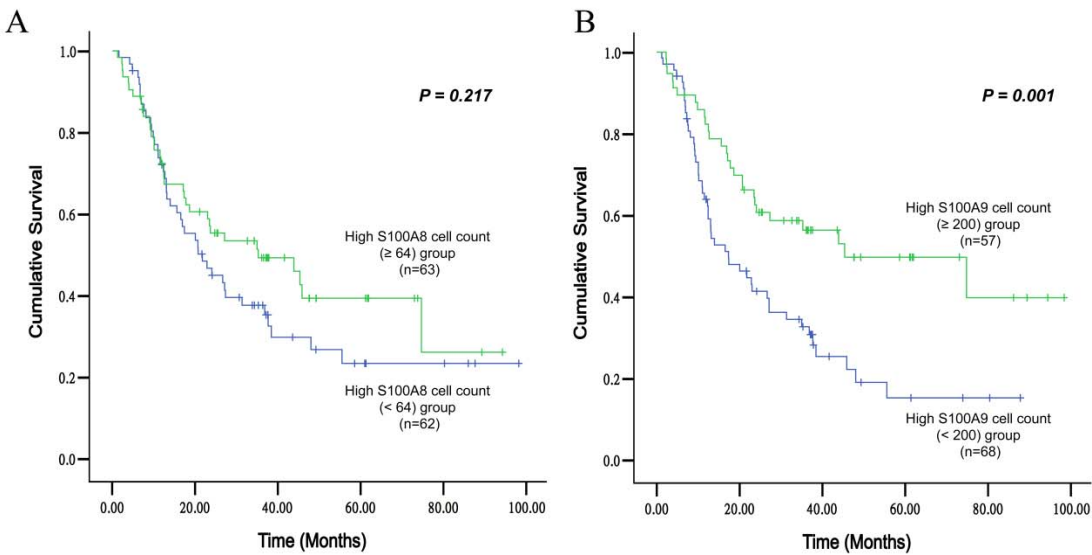

Supplement: Additional file 3 — Figure S2. High S100A9, not S100A8 cell count indicates better outcome in gastric cancer patients. (A) Kaplan-Meier analysis of overall survival for high S100A8 cell count (> = 64) group and low S100A8 cell count (< 64) group in 125 gastric cancer patients. (B) Kaplan-Meier analysis of overall survival for high S100A9 cell count (> = 200) group and low S100A9 cell count (< 200) group in the same cohort of gastric cancer patients. [file 1471-2407-12-316-S3.pdf]
