# Supplementary material for: Presence of S100A9-positive inflammatory cells in cancer tissues correlates with an early stage cancer and a better prognosis in patients with gastric cancer
Source: BMC Cancer. 2012 Jul 28;12:316. doi: 10.1186/1471-2407-12-316 (PMC3476982; doi:10.1186/1471-2407-12-316)
Supplement: Additional file 2 — Table S1. Association of S100A8-positive inflammatory cell count in cancer tissues with clinicopathological parameters in gastric cancer patients. [file 1471-2407-12-316-S2.pdf]

Table S1. Association of S100A8-positive inflammatory cell count in cancer tissues with clinicopathological parameters in gastric cancer patients.

Factor	S100A8 negative (positive cells <64)	S100A8 positive (positive cells>=64)	*P* value
Gender (%)			0.798
Male	45(72.60)	47(74.60)	
Female	17(27.40)	16(25.40)	
Age (%)*			0.000
<50	0(0.00)	39(61.90)	
50-59	0(0.00)	24(38.10)	
60-69	48(77.40)	0(0.00)	
>=70	14(22.60)	0(0.00)	
TNM stage (%)*			0.228
I	3(4.80)	9(14.30)	
II	13(21.00)	8 (12.70)	
III	22(35.50)	22(34.90)	
IV	24(38.70)	24(38.10)	
Tumor location (%)			0.871
Cardia	14(22.60)	15(23.80)	
Non-cardia	48(77.40)	48(76.20)	
Depth of mural invasion (%)*			0.903
T1	1(1.60)	2(3.20)	
T2	10(16.10)	12(19.00)	
T3	37(59.70)	35(55.60)	
T4	14(22.60)	14(22.20)	
Lymph node metastasis (%)			0.162
N0	10(16.10)	18(28.60)	
N1	23(37.10)	18(28.60)	
N2	16(25.80)	20(31.70)	
N3	13(21.00)	7(11.10)	
Distant metastasis (%)			0.088
Negative	56(90.30)	50(79.40)	
Positive	6(9.70)	13(20.60)	
Vascular invasion (%)			0.973
Negative	25(41.00)	24(40.70)	
Positive	36(59.00)	35(59.30)	
Differentiation (%)*			
Well	1(1.60)	5(7.90)	0.333
Poorly+moderately	53(85.50)	50(79.40)	
Other types	6(9.70)	7(11.10)	
Not determined	2(3.20)	1(1.60)	

* Fisher’s exact test
